# A Digital Game and School-Based Intervention for Students in Hong Kong: Quasi-Experimental Design

**DOI:** 10.2196/12003

**Published:** 2019-04-05

**Authors:** Angie KY Shum, Eliza SY Lai, Wing Gi Leung, Mabel NS Cheng, Ho Kit Wong, Sam WK So, Yik Wa Law, Paul SF Yip

**Affiliations:** 1 The Hong Kong Jockey Club Centre for Suicide Research and Prevention The University of Hong Kong Hong Kong China (Hong Kong); 2 Department of Social Work and Social Administration The University of Hong Kong Hong Kong China (Hong Kong)

**Keywords:** digital game-based learning, school-based learning, mental health, schools, students, child welfare, health promotion, follow-up studies, internet access, public health, non-randomized controlled trials

## Abstract

**Background:**

In Hong Kong, with an increasing number of children experiencing mental health issues, there is a need to not only develop innovative interventions but also develop comprehensive prevention interventions so as to reduce their anxiety symptoms and enhance their emotional management and interpersonal relationships.

**Objective:**

The aim of this study was to determine the effectiveness of *The Adventures of DoReMiFa*, an integration model of the cognitive-behavioral approach and positive psychology by using digital game–based and school-based mental health enhancement intervention to magnify the social and emotional health and well-being of the school children in Hong Kong aged 9 to 11 years.

**Methods:**

A quasi-experimental design method was used to evaluate this digital game and school-based intervention. *The Adventures of DoReMiFa* was piloted in 4 primary schools where students were allocated to either an intervention or a control group. The participants were assessed at pre- and postintervention with a 6-month follow-up measuring their mental health knowledge, levels of anxiety symptoms, positive and negative thinking, perspective-taking, and self-esteem.

**Results:**

A total of 459 primary school students from 4 primary schools participated in the study. The response rate on the questionnaires answered on the Web was up to 85.1% (391/459). Compared with the control group, the intervention group was found to have significant association with improved mental health knowledge at the time immediately after the intervention (beta=.46; *P*=.01) and in the 6-month postintervention period (beta=.66; *P*<.001); for perspective-taking, the intervention group had exhibited a significant improvement 6 months after the completion of the universal program (beta=1.50; *P*=.03). The intervention, however, was found not to be effective in reducing the rates of anxiety symptoms and negative thinking among the participating students.

**Conclusions:**

*The Adventures of DoReMiFa*, an integration of a digital game–based and school-based mental health enhancement intervention, was shown to be effective in elevating the knowledge of mental health and promoting perspective-taking in the primary school students of Hong Kong. Although there was insufficient evidence to support a reduction in symptoms of anxiety and negative automatic thoughts, the overall results were still encouraging in that a preventive effect was found, indicating that the program has the potential to enhance the mental well-being of schoolchildren. It also suggests that knowledge enhancement may not necessarily lead to behavior change, and more focused effort may be needed to achieve the translation. The implications and limitations of this study and suggestions for future research were also discussed.

## Introduction

### Childhood Mental Disorders

Mental health is defined as a state of well-being in which an individual realizes his or her own potential, can cope with the normal stresses of life, can work productively and fruitfully, and is able to make a contribution to her or his community. [1]

The US Department of Health and Human Services further emphasizes that mental health in childhood is characterized by the achievement of developmentally appropriate milestones such as effective coping and healthy interpersonal skills [2]. Although there are various specific definitions of mental health in different cultures, together, the overall concepts incorporate the social, emotional, and behavioral well-being of children and adolescents. In younger children, common signs of distress may include crying and expressing worries and fear (emotional), isolating oneself (social) or aggressive behaviors, and withdrawing from peers and other pleasurable activities (behavioral) and the like. Although many children occasionally display these symptoms, it is the frequency and severity of those symptoms that are of concern.

Internalizing problems, specifically depression and anxiety disorders, are often characterized by covert symptomatology and are therefore difficult to recognize and verbalize [3]. Epidemiological studies have shown that the prevalence rate of specific internalizing difficulties in children and adolescents, that is, depression and anxiety disorders, has been alarmingly high in the past decades, and anxiety disorders have been shown to be the most common form of mental illnesses in the youngsters [4-6]. The prevalence rate of anxiety disorders in children varies from 10% to 23%, with the lifetime prevalence rate estimated to be about 29% [6-8]. The results from another national community survey further indicated that nearly 1 in 3 American youths aged 13 to 18 years has met the criterion of an anxiety disorder [9].

### Prevalence of Childhood Anxiety in Hong Kong

In Hong Kong, the prevalence of internalizing difficulties among children and adolescents has drawn great attention to educators, clinicians, and researchers. In a prevalence study on childhood internalizing problems, Siu examined 1598 mothers of local primary school children and reported that the prevalence of internalizing difficulties among the young children was 11.4% [10]. However, the actual rate of prevalence may have been overlooked as many children may remain unidentified by their parents and teachers. In particular, owing to the strong stigmatization and discrimination in Chinese societies, despite the parents being aware of the illnesses in their children, they may tend to minimize the symptoms [11].

### Expanding Mental Health Promotion in the School Setting

Although there are many effective treatments available for childhood anxiety disorders such as cognitive behavioral therapies, medication treatments, or a combination of the two [12-14], nonetheless, many children fail to receive timely and formal treatments. Furthermore, it has been shown that half of the mental disorders found in adults start at the age of 14 years, where anxiety symptoms among adults surface even earlier, with a median age of 11 years [6]. As a result, preventive intervention may seem to be an ideal method, focusing on young children before the onset of the symptoms and diagnosable disorders. Thus, school would be an ideal platform to promote the mental health of children. During the past decades, research found that schools have become the most common point of entry and provider of mental health promotion services. It is also shown to have various advantages, including reaching out to a larger number of students and reducing common obstacles to treatment access such as time and costs [15,16].

Over the past 10 years, the Hong Kong Jockey Club Centre for Suicide Research and Prevention (The Centre) has been implementing various mental health promotion programs for primary and secondary school students in Hong Kong. In 2006, based on the cognitive-behavioral theory, the first universal school-based prevention program, *Little Prince is Depressed*, was initially developed with the aim to reduce depressive symptoms and enhance protective factors of secondary school students in Hong Kong. The results indicated a significant improvement in help-seeking attitudes and self-esteem among the students who have completed the teacher-led mental health program [17]; however, there were also considerable challenges such as a small sample size, tight teaching schedule, and low teacher efficacy and so forth. To tackle the difficulties and limitations mentioned above, in 2010, an internet-based mental health program, *Professor Gooley and The Flame of Mind* [18], was further developed. It consisted of cognitive behavioral theory and positive psychology frameworks targeted to enhance the mental well-being of adolescents. Despite the innovative design and interesting storyline, the Web-based program also encountered problems such as high dropout and low completion rates.

### Framework of This Study

Learning from past experiences, it is believed that the involvement of teachers in school programs can be decisive in motivating and engaging students to complete the programs. Furthermore, in the Web-based programs, the students are exposed to the contents of the program before each school-based lesson, which may aid to tackle the tight teaching schedule. This in turn provides more time for the teachers to guide interactive activities, for discussions and reflections during classes, and these are believed to be useful to consolidate the students’ knowledge and the skills learned. With these benefits, this study aimed to investigate into the effectiveness of integrating both school-based and digital game–based frameworks. It is believed that all students can benefit from such a form of program delivery mode and skills building program. It is predicted that the intervention effect of this program may further be strengthened through the enhancement of the children’s intrapersonal (such as self-awareness, self-esteem, and perspective-taking) and interpersonal (such as social and communication skills) functioning in schools. In addition to the elements of the cognitive-behavioral approach, this program also integrated key elements of the positive psychology perspectives to promote the mental well-being of students.

Positive psychology consists of valued subjective experiences such as well-being, contentment, hope, flow, and happiness. Its fundamental goal is to promote positive mental health in the community [19,20]. In recent years, positive education programs have become increasingly popular in schools, with an ultimate goal to enhance the well-being of students and parents as well as teachers. Various studies have been conducted to demonstrate the potential benefits of the positive education intervention programs in cultivating positive emotions, behaviors, and attitudes among the schoolchildren [21,22]. Given the significant impacts of positive education among children, key elements of the positive education model, that is, positive emotions, empathy, and gratitude, were integrated into this program. It is believed that enhanced perspective-taking skills and the exercise of gratitude could yield significant improvement in the children’s well-being. In addition, although there is no formal curriculum for mental health education in Hong Kong, some of its concepts may be brought up during homeroom or moral and civic education lessons. Nevertheless, there is still a lack of evidence-based and systematic mental health education programs for young children in the schools in Hong Kong.

Furthermore, the use of the internet has already penetrated through people’s daily lives. Many studies have shown that the internet has been increasingly utilized by health care providers to provide the latest information and interactions with their patients and the general public, in particular, among the young generation [23-26]. In addition, in view of the high-demanding curriculum and competitive teaching hours at school, a transition to a more digital learning environment seems to be necessary for the students in Hong Kong. As such, to attract the students’ interest in mental health and promote their learning in the relevant skills, a digital game–based approach was designed and integrated into the entire program. It was written into an adventurous story, and the purpose of each digital game–based lesson was used to prepare the students for the content of the school-based lesson.

### Aims of This Study

In Hong Kong, the majority of studies to date have focused on treatment interventions. This study however sought to extend the research to explore into the prevention of childhood anxiety disorders and the enhancement of the students’ mental well-being through an innovative online and offline approach. The objective was to examine the effectiveness of implementing a school-based and digital game–based intervention program with a combination of the cognitive-behavioral and positive psychology model. Through this, it aimed to reduce childhood anxiety and improve the mental well-being of students. This study has 4 specific objectives: (1) the primary objective was to compare the self-reported anxieties in an intervention condition and a monitoring condition at different time points. It was hypothesized that compared with the children in the monitoring group, the intervention group would have greater reduction in self-reported anxiety after completing the program; (2) the second objective was to examine the effectiveness of the interventions on the children’s mental health knowledge. It was hypothesized that the children in the intervention condition would evidence a greater enhancement in the relevant knowledge; (3) the third objective was to examine the children’s mental well-being. The intervention group was expected to have more positive thinking, enhanced self-esteem, and empathy skills at the postintervention and the 6-month follow-up time intervals; and (4) the final objective was to evaluate the effectiveness of digital game–based learning in equipping students with the relevant knowledge and skills. It was hypothesized that compared with the students who completed less on the Web-based learning, students who had a high completion rate (at least completed 6 modules) on the Web-based learning would result in lower rates of self-reported anxiety symptoms and better mental well-being.

## Methods

### Recruitment

#### Intervention Effect

As randomization was not preferred by the participating schools, this study is a quasi-experimental design. On the basis of their needs, suitability, and capability, the school teachers would prefer to assign all students of the same grade (ie, either Primary 4 or Primary 5) to either an intervention or a control condition. All participating schools had both intervention and control groups (ie, students of 1 grade was the intervention group and another grade in the same school was the control group). Before the baseline assessment, informed consent was obtained from all participants and their parents. The students were asked to fill out questionnaires at the preintervention stage (T0), postintervention stage, that is, 2 weeks after completion of the program (T1), and at the 6-month follow-up (T2).

#### Digital Game–Based Learning Effect

On the basis of the same group of students recruited above, further analyses were conducted to assess the efficacy of the digital game–based program. On the basis of their completion rate on the digital game–based learning, the students were divided into 2 groups. Those students who have reached the completion rate of 50% or above were in the high completion group, whereas those below 50% were in the low completion group.

### Procedure

In total, 4 primary schools completed the program in the period of 2014 to 2015. At the enrollment stage, an information seminar on the program was organized in June 2014. After the seminar, a few schools showed their interest to join the program. Invitation letters including information sheets, the program outline, and enrollment forms were then sent to the interested schools and all primary schools in Hong Kong. An initial consent was obtained from the principal of each school to invite the students, their parents, and teachers to participate in this study. On the basis of the needs, suitability, and capability of the students, the school teachers would assign the students of 1 grade as an intervention group and another grade as a control group. All the parents of the students were sent an information sheet describing the program, and an informed consent form was to be completed and returned by the parents. The consent rate was 42.50% (459/1080).

*The Adventures of DoReMiFa* was a digital game–based and school-based mental health enhancement program for Primary 4 and 5 students aged 8 to 12 years. The content was designed and developed by multidisciplinary professionals including clinical psychologists, social workers, counselors, and educators and adopted the cognitive-behavioral therapeutic approach and positive psychology as theoretical frameworks. The program consisted of 8 modules including (1) emotional competence; (2) cognitive model; (3) ABC theory; (4) problem-solving skills; (5) social skills; (6) communication skills; (7) empathy; and (8) gratitude. The program was a combination of digital game–based and school-based teaching. Altogether, the 8 modules were transformed into 11 digital game–based lessons and 8 classroom teachings. This project was supported by the Quality Education Fund of the Education Bureau of Hong Kong.

To prepare for the design of the digital game–based lessons, a few focus groups were conducted with the students, parents, and teachers separately to collect their views on mental health, the program design, and digital game learning. The digital game–based lessons were written into an adventurous story that combines the elements of a storyline, dialogues, problem-solving, challenges, mini-games, teamwork, and so on. The story was about 4 monsters, Do, Re, Mi, and Fa, who came from another planet to look for a book hidden in a primary school. The hidden book would help to improve the mental health of their fellow citizens. They would encounter 2 students, Lily and Max, after they landed on Earth to help them look for the book and solve different challenges and tasks. Each digital game–based lesson lasted about 20 min and was used to prepare the students for the content of the upcoming classroom lesson. Details of the program can be found at the official website of *The Adventures of DoReMiFa* [27]. Each student would be given a unique login name and password by their teacher during the school-based introduction lesson.

After each digital game–based lesson, a classroom lesson would follow to facilitate and consolidate the students’ learning. The classroom lessons were led by graduates or students of a master’s degree in Counseling or Counseling and Clinical Psychology or were qualified teachers who had received an 8-hour preservice training. All instructors would teach the same class throughout the 8 school-based lessons, and the class teachers were required to stay in the classroom to observe the teaching and facilitate the activities and discussions. The structure of each lesson (approximately 25 to 60 min/lesson) involved different interactive activities such as role-play and card games. Textbox 1 presents the corresponding digital game–based and school-based lessons in sequential order.

**Distribution of the digital game–based lessons and school-based teachings.**

School-based lessons:

- Lesson 1: Introduction of the program
- Lesson 2: Emotional competence
- Lesson 3: Cognitive model and ABC theory
- Lesson 4: Problem-solving skills
- Lesson 5: Social and communication skills
- Lesson 6: Empathy
- Lesson 7: Gratitude
- Lesson 8: Review

Digital game–based lessons:

- Lesson 1: Emotional competence
- Lesson 2-3: Cognitive model and ABC theory
- Lesson 4-5: Problem-solving skills
- Lesson 6-8: Social and communication skills
- Lesson 9: Empathy
- Lesson 10: Gratitude
- Lesson 11: Review

In this study, the students were assessed at 3 time intervals—before the first digital game–based lesson (T0: preintervention), 2 weeks after completion of the program (T1: postintervention), and 6 months after completion of the program (T2: follow-up). The questionnaire was self-administered and consisted of 5 parts to measure 5 outcomes of the study, including (1) anxiety; (2) mental health knowledge; (3) positive and negative thinking; (4) perspective-taking; and (5) self-esteem. All participants in the intervention group and control conditions were invited to complete the same set of questionnaires online through the official website of *The Adventures of DoReMiFa* in a similar time frame. Informed consent was sent to all students and their parents before the study and all students could opt out from the study at any time. Background information of the students such as class numbers and dates of birth were used as identifiers to match with the postintervention and follow-up tests.

### Measurements

#### The Screen for Child Anxiety–Related Emotional Disorders

The Screen for Child Anxiety–Related Emotional Disorders (SCARED) is designed to evaluate anxiety disorder symptoms of children and adolescents aged 8 to 18 years [28]. It consists of 41 items measuring 5 anxiety disorders. In this study, 9 items were used to measure the children’s Generalized Anxiety Disorder on a 3-point Likert scale (from 0=Not True or Hardly Ever True to 2=Very True or Often True). The optimal cutoff for the Generalized Anxiety Disorder subscale was 8. The whole SCARED scale had a good internal consistency (Cronbach alpha=.95) as well as its subscales (Cronbach alpha=.72 to .88) and was a suitable and useful instrument to screen for anxiety disorders in children and adolescents in a community or school setting [29-31]. A Chinese version of the scale was used in this study.

#### Mental Health Knowledge Checklist

An 11-item knowledge checklist was designed by the research team to examine the mental health knowledge of the students before and after the program. Each item on the checklist was generated from the knowledge and skills taught in the program, and all items were *True* or *False* questions. Students would score 1 point for a correct answer and 0 for an incorrect answer. A Chinese version of the scale was used in this study.

#### Children’s Automatic Thoughts Scale-Negative or Positive

The original Children’s Automatic Thought Scale-Negative/Positive (CATS-N/P) consists of 4 subscales, each has 10 items measuring negative self-statements across both the internalizing and externalizing problems of children and adolescents aged 8 to 17 years [32]. In this study, only 1 subscale, that is, personal failure, was adopted to assess the children’s negative thoughts about themselves. In addition to the personal failure subscale, another 10 positive items were added to facilitate the calculation of the state-of-mind ratios [33]. Then, corresponding to the frequency of their experience over the past week, the students were asked to rate each statement on a 5-point Likert scale from *Not at all* (0) to *All the time* (4). Higher scores reflect a greater number of negative or positive automatic thoughts. As indicated by Cronbach alpha coefficient of .79 to .95, CATS-N/P has good internal consistency and test-retest reliability [32,34]. A Chinese version of the scale was used in this study.

#### Interpersonal Reactivity Index

The original Interpersonal Reactivity Index (IRI) is a 28-item self-report measure of the students’ perspective-taking behaviors [33]. In this study, a Chinese version of the IRI (C-IRI) was adopted [35]. The items were grouped under a 3-factor model—personal distress, fantasy scale, and empathy. The empathy subscale (11 items) mostly consists of items taken from the perspective-taking and empathic concern subscales of the original IRI. In this study, the perspective-taking items (6 items) from the empathy subscale in the C-IRI were used. Furthermore, the students were asked to rate each statement on a 5-point Likert scale from 0 (Does not describe me well) to 4 (Describes me very well). Higher scores show a higher level of perspective-taking (empathy). The C-IRI was found to have acceptable psychometric properties in the Chinese context with good internal consistency (Cronbach alpha of .65 to .70) and a 2-week test-retest reliability of .68 to .83 [35].

#### Rosenberg Self-Esteem Scale

The Rosenberg Self-Esteem Scale (RSES) consists of 10 items measuring the students’ self-esteem [36]. All items were answered using a 4-point Likert scale format ranging from *Strongly disagree* (1) to *Strongly agree* (4). After reversing the scores of 5 negative items, higher scores indicate a higher level of self-esteem. This scale has good reliability and validity. Fischer and Corcoran [37] showed that the 2-week test-retest reliability was high (rs>.80) and the scale correlated significantly with other measures of self-esteem and depression and anxiety in predicted directions. The Chinese version of RSES was validated by Yeung [38] and used in this study.

### Data Analyses

#### 1. Intervention Effect

Multilevel modeling was used to analyze the data. Similar analytical approach was employed in an evaluation study on a school-based prevention program of depression [39]. A 3-level regression model was first employed to test if significant group differences (between the intervention group and the control group) existed during the pretest assessment on each outcome measure including knowledge, anxiety, negative and positive automatic thoughts, perspective-taking, and self-esteem.

To test if intervention effects existed between the 2 groups, a series of 4 level models were individually employed on each outcome. Level 1 accounted for the changes within the students on the outcome measure, whereas levels 2, 3, and 4 accounted for changes between students, between classes, and between schools, respectively. Independent variables included gender, age, time variable (T0 as the pretest [ie, baseline], T1 as the posttest, and T2 as the follow-up test), the group variable (intervention and control), and their interaction term (ie, time×group). The interaction term *T1* × group examined the differences across 2 groups immediately after completion of the program. A statistically significant coefficient indicated the changes of scores varied across groups, suggesting an existence of interaction effects. Similarly, the interaction term *T2* × group examined the differences across groups during the follow-up assessment and was employed to test whether there was an interaction effect after 6 months of completion of the program.

If significant interaction effects were found in both *T1* × and *T2* × groups, this would indicate significant persistence of intervention effects, suggesting effects were found immediately and have persisted for 6 months after completion of the program. If, however, significant interaction effects were only found in the *T1* × group but not in the *T2* × group, this would indicate significant immediate intervention effects but diminish afterward. On the contrary, if significant interaction effects were only found in the *T2* × group but not in the *T1* × group, this would indicate no intervention effect right after completion of the program but a significant 6-month delayed intervention effect.

#### 2. Digital Game–Based Learning Effect

Further analyses on the intervention group were conducted to determine the effects of Web-based programs in terms of the number of Web-based modules completed at the end of the implementation of the program. There were 11 modules in total. On the basis of the number of completed modules, the students from the intervention group were further categorized into 2 groups (below and above the completion rate of 50%, that is, whether completed at least 6 modules). Multilevel analyses, as described, were then used to determine the completion levels of the Web-based modules in relation to the improvement in outcome measures within the intervention group at different assessment intervals. A significant coefficient of the interaction term time×group would suggest that if sufficient modules were completed by the students, they would have experienced relatively greater improvement in a certain outcome measure.

All analyses in 1. and 2. above were performed by PROC MIXED in the statistical program SAS (Statistical Analysis System) version 9.3 for Windows by SAS institute [40], and all statistical significances were determined at a 5% level.

## Results

### Intervention Effect

#### Demographic Information of the Participants

**Figure 1:**
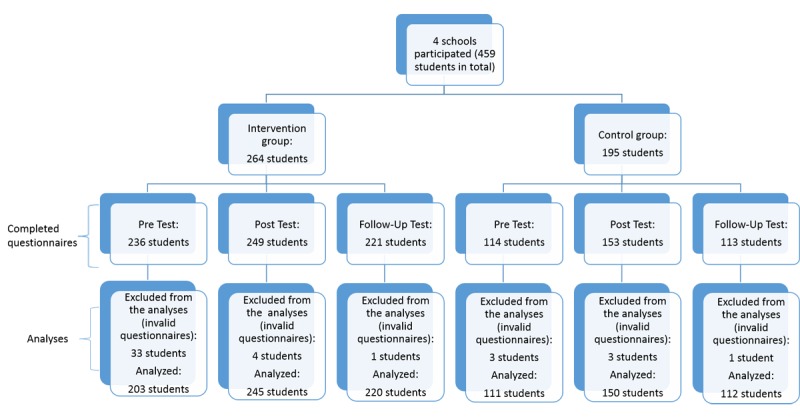
Completed questionnaires analyses. Participants who only completed one assessment point and questionnaires with sloppy answers were excluded from the analyses. As some students did not initially complete the pretest questionnaires, more participants completed the posttest questionnaires than the pretest questionnaires in the analyses but managed to complete both post and follow-up tests.

The participants were 459 children aged 8 to 12 years from 4 primary schools in Hong Kong. It included 3 coeducational schools and 1 girls’ school. Overall, 264 and 195 students were in the intervention group and control group, respectively; 232 were Primary 4 students (50.6%; 232/459) and 227 (49.4%; 227/459) were Primary 5 students. The mean age of the students was 9.53 years with an SD of 0.717 in the intervention group and 9.48 years in the control group (SD 0.64). Entries by students who showed the same response for all items across 3 measures or above and those who only completed 1 out of the 3 time points were excluded from the analyses. In the end, 314, 395, and 332 students were put into the analyses in T0, T1, and T2, respectively. Please refer to Figure 1 for the flow of data exclusion.

### Differences at the Pretest Assessment

At the pretest assessment, no significant differences were found between the 2 groups across knowledge, anxiety, negative automatic thoughts, perspective-taking, and self-esteem. Positive automatic thoughts, however, were found to have a significant difference during baseline assessment across the 2 groups.

Multimedia Appendix 1 summarizes the scores of all outcomes measured during pretest, posttest, and follow-up assessments between the intervention and control groups.

Results of the estimated interaction effects from multilevel modeling are shown in Multimedia Appendix 2. Significant results were found in the participants’ mental health knowledge and perspective-taking (empathy). The intervention effects were strong in mental health knowledge. A significant intervention effect was found immediately after completion of the program. The students showed significant enhanced mental health knowledge in the intervention group (beta=.46; *P*=.01). Follow-up assessments showed a very prominent persistent effect of intervention where further improvement on mental health knowledge was found after 6 months of completion of the program (beta=.66; *P*<.001). As for perspective-taking, no significant intervention effect was found immediately after completion of the program; however, a significant delayed intervention effect was found at T2, 6 months after completion of the program (beta=1.50; *P*=.03). No significant intervention effects were found in the outcome measures of anxiety, positive and negative automatic thoughts, and self-esteem among the participants immediately and 6 months after completion of the program. Although there was a slight increase of the mean score in the outcome measure of anxiety level at T2 of the intervention group, all scores were within the normal range. Although no intervention effects were found, on the whole, positive improvements and trends were shown by the means at each time point of negative and positive automatic thoughts and self-esteem.

### Digital Game–Based Learning Effect

#### Demographic Information of the Participants

A total of 264 students from the intervention group were divided into high and low completion groups. Of the 11 modules, 182 (68.9%; 182/264) students reached the completion rate of 50% or above (high completion group), whereas 82 (31.1%; 82/264) completed 50% or below (low completion group). Overall, 139 (76.4%; 139/182) females and 43 (23.6%; 43/182) males were in the high completion group, whereas 43 (52.4%; 43/82) females and 39 (47.6%; 39/82) males were in the low completion group.

No significant differences were found on each outcome measure between the students of the high and the low completion of modules at the pretest assessment (the third far right column), which suggest that students’ participation in the Web-based modules was not likely to be associated with their pretest performances.

Multilevel modeling was then applied to identify the existence of any significant differences in the improvement on each outcome measure between students with higher and lower completion of Web-based modules since pretest, at posttest, and follow-up assessments. Results of the estimated effects are given in Multimedia Appendix 2 (the far right 2 columns). When compared with the lower completion group (ie, completion rate below 50%), among the higher completion group (ie, completion rate above 50%), and immediately after completion of the program, significant improvements were found on the students’ mental health knowledge (beta=.51; *P=*.04) and positive automatic thoughts (beta=3.32; *P*=.007). However, no significant differences in improvement between the higher and lower completion groups were found on the 6 outcome measures 6 months after completion of the program.

## Discussion

### Principal Findings

This study showed that the combination of school-based and digital game–based program, *The Adventures of DoReMiFa*, was effective in promoting the students’ mental health knowledge, positive automatic thoughts, and perspective-taking in Primary 4 and 5 students in Hong Kong. Specifically, when compared with the control group, the results showed an immediate and sustained enhancement on the students’ mental health knowledge after implementation of the program. Also, the program was likely to act as a buffer against deterioration of the students’ empathetic skills (perspective-taking) 6 months after completion of the program, indicating a delayed intervention effect on this protective factor. Additional exploration was also conducted to investigate into the impact of the high versus low completion rate of the digital game–based intervention program among students who were intervened. When compared with those in the low completion rate group, it was found that students in the high completion rate group showed better results in mental health knowledge and enhanced positive automatic thoughts immediately after implementation of the program; however, a sustained effect was not found.

The study also provided sufficient evidence on the effectiveness of utilizing both digital game–based and school-based methods in delivering mental health enhancement programs in Hong Kong. It was shown to be particularly effective in the mental health knowledge of the intervened students even 6 months after implementation of the program, suggesting a prominent continued effect of enhancement in mental health knowledge among the students. This further indicated that the students had advanced their understanding of various concepts and the skills taught in the program, including emotional competence, problem-solving, communication skills, empathy, cognitive model, ABC theory, and so on. Furthermore, the results of this study also found a delayed intervention effect on perspective-taking, a measure on empathy. The effect, however, was only found to be prominent 6 months after implementation of the program but not immediately. Besides, it was surprising that no instant or sustained effects were found in empathetic skills; however, when compared with the control group, the students in the control group showed a significant deterioration of perspective-taking in 6 months. This phenomenon may imply that this program could act as a buffer toward the reduction of empathy among the students in the intervention group, that is, students in the intervention group maintained their level of empathy throughout the program. Another explanation is that the taught skill, empathy, may require longer duration to be nurtured among the students. In other words, it is possible that not only the students might need extra time to understand the concept but also time to transfer it into practice, which then in turn may enhance their thorough understanding on empathy. No significant effects, negative automatic thoughts, and self-esteem, however, were found in the measures of anxiety and well-being. Possible explanations could be the students in this study were not randomly assigned into intervention and control groups, and also, the duration of the intervention period was short. Therefore, future studies could look into the optimal intervention duration that best benefits the students in Hong Kong and make further improvement on the research method for this type of study to be more effective.

Moreover, the project team intended to investigate into the changes among the students on each individual outcome, and although not many tests were conducted, significant results were found. Multiple comparisons were further conducted to investigate into the prominence of the proposed outcome measures. In both the program intervention and digital game–based learning effect, among the known significant effects of mental health knowledge, perspective-taking, and positive automatic thoughts, after the program implementation, a relatively strong sustained intervention effect was observed on the mental health knowledge of the Primary 4 and 5 students. In addition, a strong significance on positive automatic thoughts was also observed in the students who were in the high completion rate group of the digital game–based program. Thus, this further shows the robustness of the effectiveness of such a digital game–based and school-based mental health enhancement program.

Although numerous studies have shown that universal school-based programs are effective in yielding positive outcomes to reduce anxiety-related symptoms in children and adolescences [41,42], this study failed to show a significant reduction in anxiety among the intervened students. One explanation is that the program was entirely led by mental health professionals rather than by school teachers. Previous research studies have shown positive impacts on the program effectiveness in reducing anxiety symptoms when mental health programs were led by teachers rather than by mental health professionals. Recently, a local study conducted in The Centre by Lai et al [43] also found that teacher-led mental health prevention programs yielded better outcomes in reducing anxiety symptoms in students than professional-led groups. It was thus suggested that, in the long-run, classroom teachers will achieve better sustainability in school-based mental health prevention programs as they have sufficient amount of time with the students and already have pre-existing rapport built throughout the school year [44]. Hence, in view of these research studies, with the aim to deliver the best quality program to promote well-being and reduce anxiety and depressive symptoms, future studies should utilize classroom teachers as school-based mental health prevention program instructors. Another possible explanation could be due to this program being a universal program where the mean scores of the students’ anxiety levels were within the normal range before the implementation program; therefore, it is of no surprise that there were no significant reductions of this outcome measure after the program implementation. Similar results were also found in a previous study suggesting that the students who had more depressive symptoms before the program implementation showed better improvement in cognitive restructuring skills and support-seeking behaviors after the program [17]. Thus, future studies may investigate into the intervention effect between the students who score higher in the anxiety level before the program and in the control study. All the above limitations mentioned indicate that perhaps the implementation of a mental health program solely in 1 school year may not be effective in improving the students’ mental health if the school environment remains unchanged. With the new era of the whole school approach and implementation of mental health programs in the school curriculum [45], it is of utmost importance to promote the whole school approach on the mental well-being among schools. Hence, it is worthwhile investigating into the effectiveness of delivering a high-quality mental health program in the school curriculum. Through this investigation and implementation, the research team anticipates yielding positive and promising significant results in improving the mental health of the students in Hong Kong.

Moreover, this program exhibits positive impacts and effectiveness in that it has provided more evidence on the effectiveness of the universal mental health enhancement program in the school setting. In addition, moving onto a preventive mental health model, many overseas researchers have been investigating into the effectiveness of increasing the social, emotional, and psychological skills in the students via mental health enhancement programs in school. A great example conducted by Seligman et al [46] has brought tremendous impacts on the students by using positive psychology in the school setting. This study further showed this positive phenomenon through the application of positive psychology as well as cognitive behavioral theory in schoolchildren. All these studies successfully showed promising results in supporting and nurturing the mental health of students in a holistic approach and under the classroom setting [46-48]. In addition, this study further suggested that schools can be a great platform for students to learn about mental health–related knowledge. Furthermore, primary school students encounter many ups and downs within the school setting, suggesting that school-based programs on mental health promotion designed according to the school context may be beneficial to the students, as this would assist them in learning and applying the taught techniques and theories to their daily situations.

To the best of the authors’ knowledge, thus far, no study has investigated into the effectiveness of utilizing the mode of delivery in conjunction with school-based and digital game–based interventions to implement mental health school programs in Hong Kong. Recent evidence showed promising results on the efficacy of computer- and internet-based cognitive behavioral treatments among the youth with anxiety and depression, indicating that online-based mode of delivery could be an alternative to traditional face-to-face treatments [13]. Moreover, previous research on school-based or digital game–based programs has also demonstrated their own plausible proficiency and effectiveness. The former showed decreased levels of depression, anxiety and stress, and enhanced mental health knowledge and positive attitudes toward mental illnesses among the adolescents, whereas the latter showed good multimedia capabilities, far-reaching abilities, and timeless and effortless accessibilities [39,49]. Nonetheless, a major limitation was found on the attrition of users while implementing the digital game–based program alone. A possible explanation is that the program was a self-motivated learning program where students were asked to complete without the involvement of teachers. It was reported that an overall 57% attrition rate was found in the systematic review and meta-analysis of the digital game–based program [50], indicating that dropouts are common during the implementation of the internet-based program and thus suggesting that it may not fully excel when implemented on its own. However, the attrition rate can be reduced when the programs were supported by therapists or with additional administrative support, demonstrating that human support will enhance the effectiveness of such programs. To address the engagement rate of the digital game–based programs, this study has incorporated mental health professionals as the human support of the program. The results revealed that the intervened students in the high completion group showed significantly higher scores in mental health knowledge and positive automatic thoughts than the students in the low completion group. This implied that a positive impact was found where a higher engagement rate of the digital game–based program was linked to better outcomes of the students’ knowledge on mental health and the thinking processes. Moreover, with the fast-growing pace of the internet in the past decades, delivering a mental health program as a digital game–based intervention may in fact be a cost-effective intervention in promoting mental health. This is so as this program can be delivered on the medium of the internet with no additional costs despite the increased number of beneficiaries. Furthermore, the digital game platform can be sustained and maintained on the internet server with no extra costs, meaning that the Web-based program can be used continuously after the program implementation. Also, as stated by the World Health Organization [51], depression and anxiety have cost a significant economic impact on the global economy, where it is believed that good utilization of the internet intervention may perform part of the preventive work on anxiety and depression, in turn minimizing the economic cost due to mental illnesses and unemployment. Nevertheless, e-engagement work is still challenging, requiring much effort and research into understanding how it can most benefit the students. Future studies should therefore focus on investigating into what additional components should be added into the program to enhance efficacy, adherence, and engagement of such modes of delivery.

### Limitations

Few limitations of this study were identified for future research and direction. First, no significant improvements were found in the outcome measures of anxiety, negative automatic thoughts, and self-esteem. The possible reasons could be the program was not led by teachers, had limited and inconsistent research time for questionnaire completion, and also, the teaching time was short, thus limiting the students’ learning skills and consolidation. Furthermore, it can be observed that solely implementing a mental health program may not be effective in promoting mental health and reducing the symptoms of anxiety in the students. Second, despite further analyses of the low and high digital game–based completion levels being compared between the 2 groups, the conducted statistical analyses may not specifically reveal the effectiveness of the digital game–based element of the program. Possible explanation of the results could be due to the integration model of the school- and digital game–based programs. Third, utilizing both digital game–based and school-based modes of delivery indeed showed a better engagement rate of the online platform rather than solely implementing the digital game–based program alone; however, over a quarter of the students showed low engagement in the digital game–based program. This also indicated that there was a lack of investigation into the students’ acceptability and confidence toward the presented mode of program delivery. Fourth, this study is a quasi-experimental design where a limitation would be the lack of randomization between the control and intervention groups. Finally, this study only gathered 1 posttest immediately after the program implementation, where the long-term and sustainability effects of such a program were not captured.

### Future Direction

Future research can aim to conduct a longitudinal study to best capture the effects of school-based and digital game–based mental health programs across a longer period of time, with consistent and longer class periods across various schools. In addition, further investigation will be needed to look into the optimal intervention duration that will best benefit the students such as the number of school-based and digital game–based lessons. Also, with the aim to enhance program quality and provide continuous support to the students, future programs should consider training classroom teachers to lead the program instead of employing mental health professionals. Furthermore, it has been suggested to give the students sufficient amount of time to complete the questionnaires to best capture any changes in the outcome. In response to the above constraints from the schools, future studies should focus on implementing mental health programs in the school curriculum to allow higher quality and more dosage of mental health programs in the schools in Hong Kong. To assess the effectiveness of digital game–based intervention, future studies should also include an intervention group with students who solely engage in the Web-based component of the program. With regard to enhancing the engagement of the digital game–based intervention among the students, the research team will further look into additional components that would reduce the attrition rate of students so as to increase the efficacy, adherence, and engagement of such a mode of program delivery. Moreover, adopting a similar approach conducted by Poder et al [52], a thorough investigation will be conducted to access the students’ acceptability and confidence level toward the presented mode of program delivery. In relation to the randomization between the control and intervention groups, future research in this area may incorporate a propensity score matching method into the studies. As already mentioned, the long-term effect and sustainability of the effectiveness of the program will be examined in future studies where more than 1 posttest will be conducted to examine the persistence of knowledge and skills gained.

### Conclusions

In this study, *The Adventures of DoReMiFa*, an innovative and integrated model of a school-based and digital game–based mental health promotion program, used as a universal preventive strategy, was shown to be effective in the enhancement of the students’ mental health knowledge and the maintaining of the standard of perspective-taking. This further demonstrated that early intervention and preventive programs for mental health promotion in schools were an effective way in educating the students on the importance of good mental well-being. This study also reinforces that such programs and practices should disseminate across various schools in Hong Kong so as to promote the well-being of the students and administer adaptive skills in their daily encounters. Nonetheless, to achieve a behavior change, in addition to knowledge gain, more effort may be needed. Furthermore, at times, other complimentary efforts are needed to make some standalone programs more effective.

## Appendix

Multimedia Appendix 1 Pretest, posttest, and follow-up mean scores (SDs) by group, and tests of the intervention effects from multilevel modeling. Remarks: 1 - There was an inconsistent number of students (N) across each outcome measure. Students who completed 2 time points or more on each outcome measure were put into the analyses. 2 - According to Figure 1, a total 314 students’ information was in T0, which is inconsistent with the number of students shown in this table. On top of the 314 students who were put into the analyses, the remaining samples were those who did not complete T0, but completed T1 and T2.

Multimedia Appendix 2 Pretest, posttest, and follow-up mean scores (SDs) by the number of completed Web-based modules among the students in the intervention group, and tests of the intervention effects from multilevel modeling. Remarks: 1 - There was an inconsistent number of students (N) across each outcome measures. Students who completed 2 time points or more on each outcome measure were put into the analyses. 2 - According to Figure 1, a total of 203 students’ information was in T0, which is inconsistent with the number of students shown in this table. On top of the 203 students who were put into the analyses, the remaining samples were those who did not complete T0, but completed T1 and T2.

## Abbreviations

CATS-N/P: Children’s Automatic Thoughts Scale-Negative or Positive
C-IRI: Chinese-Interpersonal Reactivity Index
IRI: Interpersonal Reactivity Index
RSES: Rosenberg Self-Esteem Scale
SCARED: Screen for Child Anxiety–Related Emotional Disorders
