# Supplementary material for: A Digital Game and School-Based Intervention for Students in Hong Kong: Quasi-Experimental Design
Source: J Med Internet Res. 2019 Apr 5;21(4):e12003. doi: 10.2196/12003 (PMC6473212; doi:10.2196/12003)
Supplement: Multimedia Appendix 1 [file jmir_v21i4e12003_app1.pdf]

Table 2

			Control ("Ctrl")			Intervention ("Intv")			Beta coefficients		
**Outcome Measures**	Range	N	T0	T1	T2	T0	T1	T2	Baseline differences	T1*Intv (p-value)	T2*Intv (p-value)
**Knowledge**	0-11	397	7.73	7.67	7.95	7.52	7.95	8.36	-0.16	0.46[a]	0.66[aaa]
			(1.00)	(1.26)	(1.26)	(1.34)	(1.26)	(1.30)		P=.01	P<.001
**Anxiety**	0-18	396	7.05	6.33	6.57	6.15	5.95	6.49	-0.72	0.53	1.00
			(4.41)	(4.07)	(4.18)	(4.13)	(4.20)	(4.15)		P=.28	P=.07
**Thoughts**											
**Negative**	0-40	396	12.25	11.34	11.17	11.93	10.41	10.31	-0.10	-0.37	0.33
			(7.44)	(7.67)	(7.47)	(8.13)	(7.67)	(7.75)		P=.66	P=.71
**Positive**	0-40	396	20.75	21.82	23.00	23.04	23.60	23.46	2.41*	0.00	-1.54
			(7.46)	(8.14)	(7.22)	(8.26)	(7.36)	(7.62)		P>.99	P=.13
**Perspective-taking**	0-24	391	16.74	15.72	15.17	16.63	16.74	16.70	-0.07	1.03	1.50[a]
			(4.79)	(4.36)	(3.89)	(5.33)	(4.52)	(4.30)		P=.08	P=.03
**Self-esteem**	10-40	391	27.99	28.87	29.10	28.79	29.15	29.43	0.79	-0.56	-0.91
			(5.61)	(4.80)	(4.95)	(4.79)	(5.00)	(5.43)		P=.29	P=.15

$^{a}p < .05$; $^{aa}p < .01$; $^{aaa}p < .001$
