# Supplementary material for: A Digital Game and School-Based Intervention for Students in Hong Kong: Quasi-Experimental Design
Source: J Med Internet Res. 2019 Apr 5;21(4):e12003. doi: 10.2196/12003 (PMC6473212; doi:10.2196/12003)
Supplement: Multimedia Appendix 2 [file jmir_v21i4e12003_app2.pdf]

Table 3

			Completion rate below 50% (low)			Completion rate above 50% (high)			Beta coefficients		
Outcome Measures	Range	N	T0	T1	T2	T0	T1	T2	Baseline differences	T1* High (p-value)	T2* High (p-value)
Knowledge	0-11	248	7.50	7.58	8.19	7.53	8.12	8.45	-0.04	0.51[a]	0.25
			(1.33)	(1.12)	(1.15)	(1.34)	(1.30)	(1.37)		P=0.04	P=0.30
Anxiety	0-18	248	6.31	6.04	6.52	6.09	5.90	6.47	-0.07	0.04	0.05
			(4.48)	(4.49)	(4.53)	(3.99)	(4.07)	(3.94)		P=0.94	P=0.94
Thoughts											
Negative	0-40	248	12.56	11.12	10.95	11.68	10.07	9.95	-1.39	-0.27	-0.18
			(8.68)	(8.36)	(8.82)	(7.92)	(7.31)	(7.09)		P=0.82	P=0.87
Positive	0-40	248	22.49	21.27	22.89	23.26	24.72	23.78	0.22	3.32[aa]	0.45
			(8.33)	(7.68)	(8.52)	(8.26)	(6.95)	(7.08)		P=0.007	P=0.73
Perspective-taking	0-24	245	16.37	16.02	16.15	16.78	17.04	17.01	-0.33	0.51	0.32
			(5.50)	(4.60)	(4.48)	(5.29)	(4.44)	(4.18)		P=0.51	P=0.73
Self-esteem	10-40	245	27.86	27.88	29.32	29.22	29.71	29.49	1.10	0.52	-1.11
			(5.26)	(5.15)	(6.07)	(4.58)	(4.82)	(5.07)		P=0.47	P=0.17

[a]$p < .05$; [aa]$p < .01$; [aaa]$p < .001$
